# miR-423-5p Regulates Skeletal Muscle Growth and Development by Negatively Inhibiting Target Gene SRF

**DOI:** 10.3390/genes15050606

**Published:** 2024-05-10

**Authors:** Yanqin Pang, Jing Liang, Jianfang Huang, Ganqiu Lan, Fumei Chen, Hui Ji, Yunxiang Zhao

**Affiliations:** College of Animal Science and Technology, Guangxi University, Nanning 530004, China; shanxi18404985210@163.com (Y.P.); liangjing@gxu.edu.cn (J.L.); 2895048136@163.com (J.H.); 19186069409@163.com (G.L.); fumeichen@gxu.edu.cn (F.C.); pyq98502@163.com (H.J.)

**Keywords:** *Guangxi Bama miniature pig*, microRNA, miRNA-423-5p, serum response factor, muscle development

## Abstract

The process of muscle growth directly affects the yield and quality of pork food products. Muscle fibers are created during the embryonic stage, grow following birth, and regenerate during adulthood; these are all considered to be phases of muscle development. A multilevel network of transcriptional, post-transcriptional, and pathway levels controls this process. An integrated toolbox of genetics and genomics as well as the use of genomics techniques has been used in the past to attempt to understand the molecular processes behind skeletal muscle growth and development in pigs under divergent selection processes. A class of endogenous noncoding RNAs have a major regulatory function in myogenesis. But the precise function of miRNA-423-5p in muscle development and the related molecular pathways remain largely unknown. Using target prediction software, initially, the potential target genes of miR-423-5p in the *Guangxi Bama miniature pig* line were identified using various selection criteria for skeletal muscle growth and development. The serum response factor (*SRF*) was found to be one of the potential target genes, and the two are negatively correlated, suggesting that there may be targeted interactions. In addition to being strongly expressed in swine skeletal muscle, miR-423-5p was also up-regulated during C2C12 cell development. Furthermore, real-time PCR analysis showed that the overexpression of miR-423-5p significantly reduced the expression of myogenin and the myogenic differentiation antigen (*p* < 0.05). Moreover, the results of the enzyme-linked immunosorbent assay (ELISA) demonstrated that the overexpression of miR-423-5p led to a significant reduction in *SRF* expression (*p* < 0.05). Furthermore, miR-423-5p down-regulated the luciferase activities of report vectors carrying the 3′ UTR of porcine *SRF*, confirming that *SRF* is a target gene of miR-423-5p. Taken together, miR-423-5p’s involvement in skeletal muscle differentiation may be through the regulation of *SRF*.

## 1. Background

The change in skeletal muscle fiber is the most important factor affecting the quality traits of livestock meat. Knowing how to produce more and better meat has always been the focus of research related to livestock. As the main meat and protein supplier in China, the study of pork production is crucial to studying the mechanism of skeletal muscle growth and development. However, the growth and development of skeletal muscle is a very complex biological process, which is regulated by many factors [1]. Previous studies have found that microRNAs (miRNAs), as a new class of regulatory factors, play an extremely important role in the growth and development of skeletal muscle. In addition, miRNAs act via targeting the 3′-untranslated region (3′-UTR) of a gene to repress their translation or to degrade mRNA [2,3]. MiRNAs primarily bind to the 3′ UTR of target genes through complete or incomplete pairing, exerting inhibition on the target gene at both the mRNA and protein levels. Consequently, they participate in various life activities, such as growth and development [4,5], cell proliferation, differentiation [6], and apoptosis, as well as the onset of diseases and tumors [7]. The majority of miRNAs simultaneously target multiple mRNAs [8]. Furthermore, certain miRNAs target the same mRNA, potentially resulting in a synergistic effect [9]. In multicellular organisms, to ensure the integrity of organism development, many miRNAs collaborate to precisely regulate each developmental stage.

The rapid and accurate prediction and identification of target genes are crucial for studying the function of microRNAs. Currently, methods such as fluorescence quantitative PCR and Western blot are commonly used to detect changes in mRNA and protein levels in cells following miRNA transfection or knockout, enabling the determination of the relationship between miRNAs and their target genes. While these methods provide high accuracy in identifying miRNA target genes, they have limitations in pinpointing miRNA target sites.

The most prevalent method for identifying miRNA target sites is the double luciferase reporter gene assay. This method involves constructing a dual luciferase expression vector, where the 3′ UTR of the target gene is cloned in vitro and connected downstream of the firefly luciferase gene vector in the dual luciferase vector at the BamHⅠ and XhoⅠ cloning sites. Subsequently, the constructed target vector and the overexpressed miRNA vector are co-transfected into cells, and the activity of the dual luciferase is measured. To further refine the analysis, mutant recombinant plasmids are constructed, where the binding site of the target gene’s 3′ UTR is knocked out. These mutant plasmids are then transfected into cells along with the dual luciferase reporter vector, and the activity of the dual luciferase is assessed. By analyzing the activity of both luciferases, researchers can determine the presence of miRNA target sites.

In recent years, numerous reports have been published on the regulation of miRNAs on skeletal muscle growth and development. Some muscular-specific miRNAs have been found, such as miR-1, miR-133, and miR-206, which are specifically and highly expressed. They act as crucial regulators in the development of skeletal muscles [10], with their expression being governed by transcription factors, including *SRF* [11], myogenic differentiation antigen (*MyoD*) [12], myogenin [13], and *MEF2* [14]. *SRF* is a member of the transcription factor family of *MADS (MCM1*, *ENS*, *SRF*) frame and is involved in regulating the expression of multiple genes. It plays a very important role in the differentiation of skeletal, smooth, and myocardial muscle types [15,16,17]. It has further been discovered that *SRF* is not only an important transcription factor for muscle-specific gene expression and postnatal skeletal muscle growth but also serves as an important prerequisite factor for initiating muscle differentiation [18]. Holstein et al. [19] found that miR-486-5p promoted the proliferation of myoblasts by inhibiting the expression of *SRF*. In addition, *SRF* may interact with some muscle genes and myogenic regulatory factors, which can not only directly interact with *MyoD* and *MyoG* but also regulate the expression of *MyoD* gene family members at the transcriptional level, thereby regulating muscle growth and development [20,21].

The miR-423 family can produce two types of mature bodies: miR-423-3p and miR-423-5p. As one of the mature bodies of miR-423, miR-423-5P is involved in many important biological processes. The miR-423-5p/PAK6 signaling pathway may serve as both a diagnostic and prognostic biomarker and also as a potential therapeutic target for managing malignant tumors [22]. In addition to its association with tumorigenesis, previous studies have demonstrated that miR-423-5p may serve as a biomarker in clinical diagnostics for heart failure. Additionally, it plays a significant role in the regulation of insulin resistance in patients with type 2 diabetes. McDaneld et al. [23] found that the expression level of miR-423-5p was the highest in piglets. The transcriptome of miRNA in porcine muscle tissue was thoroughly studied by applying a deep sequencing method, and the highly expressed miRNA is involved in the development and regeneration of skeletal muscle [24]. Despite significant advancements in prior research, much remains to be understood regarding the regulatory functions of miR-423-5p in the development of skeletal muscle.

The goal of the present study was to investigate the spatio-temporal expression of microRNA-423-5p and predict its target genes related to muscle development and perform functional verification so as to preliminarily reveal the molecular mechanism of miR-423-5p’s involvement in muscle growth and development. In this study, we first analyzed the expression of miR-423-5p in different tissues of adult *Guangxi Bama miniature pigs* and the changes in C2C12 cell differentiation after the overexpression or inhibition of miR-423-5p. Based on the potential relationship between miR-423-5p and tissue expression, the target genes of miR-423-5p were further predicted. The results of dual luciferase reporter gene detection showed that miR-423-5p could target 3′ UTR of the SRF gene. These results are helpful in further understanding the regulatory mechanism of the involvement of miR-423-5p in skeletal muscle development, and they provide a theoretical basis for the molecular improvement of pork traits in the future. At the same time, the findings also have reference significance for medical research on muscle atrophy and other diseases.

## 2. Materials and Methods

### 2.1. Animal and RNA Extraction

Four *Guangxi Bama miniature pigs* aged 0 month, 1 month, 2 months, 3 months, and 6 months (male-to-female ratio of 1:1) were obtained from the Bama Miniature Pig Breeding Center of Guangxi University. All the pigs were subjected to a small intensive feeding mode, the livestock house was warm in winter and cool in summer, and intelligent equipment was installed to prevent the occurrence of livestock stress.

The tissue types included skeletal muscle, heart, liver, spleen, brain, and kidney. All experimental procedures in this study were performed under a protocol approved by the Institutional Animal Care and Use Committee (IACUC). Total RNAs were extracted by the phenolchloroform extraction method. Additionally, cDNA synthesis was conducted using a Mir-X™ miRNA First-Strand Synthesis Kit and a PrimeScript™ RT Reagent Kit with gDNA Eraser following the provided instructions (Perfect Real Time; Dalian TaKaRa Biological Engineering Co., Ltd., Dalian, China).

### 2.2. Target Prediction and Kyoto Encyclopedia of Genes and Genomes (KEGG) Pathway Analysis

Because the current target prediction website cannot directly provide the predicted target genes of pigs and given the high homology between pig sequences and those of humans (Homo) and mice, with some sequences being identical, the Targetscan, PicTar, and miRanda databases were utilized to predict the target genes of miRNA-423-5p in humans and mice. Consequently, the intersecting target genes of miRNA-423-5p in both humans and mice were identified as the final candidate target genes.

To determine the biological function of miR-423-5p in the regulation of myogenesis, the potential targets were predicted by target detection software such as Targetscan 7.0 (http://www.targetscan.org/, accessed on 24 March 2024) and miRanda 1.9 (http://www.microrna.org/microrna/home.do/, accessed on 24 March 2024). The predicted genes were classified according to KEGG functional annotations (http://www.genome.jp/kegg/pathway.html/, accessed on 24 March 2024).

### 2.3. Design and Synthesis of Quantitative Primers

The mature sequence of porcine miR-423-5p was sourced from the miRNA information database at http://www.mirbase.org/ (accessed on 24 March 2024). A miRNA-specific forward primer was designed, and SnU6 was employed as the internal reference gene. Quantitative primers were designed based on the sequence information of SRF available on NCBI, and β-actin served as the internal reference gene. The primer sequences are detailed in Table 1 and were synthesized by the TAKARA Company (Beijing, China).

### 2.4. PCR Amplification of Pre-miR-423-5p, Product Recovery, and Simple Connection of pMD-19T

The pre-miR-423-5p sequence was amplified using the DNA template of the Bama miniature pig genome. The reaction system was as follows: 2 × Ex Taq: 25 μL, forward primer (10 μM): 1.5 μL, reverse primer (10 μM): 1.5 μL, template DNA: 2.5 μL, and ddH_2_O: 19.5 μL. The reaction procedure was as follows: 94 °C for 2 min, 94 °C, 61 °C, and 72 °C for 30 s each, after 30 cycles, 72 °C for 5 min, and the last 4 °C was maintained forever. The PCR final product was detected by 1% agarose gel electrophoresis. The PCR products were recovered using a Biospin glue recovery kit from the Hangzhou Bori Company. The recovered pre-miR-423-5p target fragment was connected to PMD-19T Simple Vector, the connection reaction liquid was mixed, and the connection was made at 16 °C overnight.

### 2.5. Plasmid Extraction, Double Digestion, and Purification

Following the instructions provided by the Tiangen (Beijing, China) Co, Ltd., for their Plasmid Extraction Kit, the extracted plasmid’s purity was assessed using a nucleic acid analyzer, focusing on the 260/280 and 260/230 ratios, and it was named PMD-19T-miR-423-5p. The PMD-19T-miR-423-5p recombinant plasmid and pLV plasmid underwent double digestion with QuickCut™ Nhe I and QuickCut™ BamHⅠ. Following purification, electrophoretic detection, and the purification of the double enzyme digestion products of the PMD-19T-miR-423-5p recombinant plasmid and pLV plasmid, the products were ligated overnight using T4 ligase in a water bath set to 16 °C.

### 2.6. Cell Culture and Transfection

Next, C2C12 cells were cultured in growth medium (GM) consisting of DMEM (Gibco, Grand Island, NY, USA) supplemented with 10% FBS (Hyclone Laboratories, Inc., Logan, UT, USA). Upon reaching approximately 90% confluence, the medium was switched to differentiation medium (DM), and culturing continued for 5 days. Additionally, the C2C12 cells were transfected with 50 nM miR-423-5p mimic/mimic-negative control (miNC) or 100 nM miR-423-5p inhibitor/inhibitor-negative control (inNC) (Applied Ribobio, Guangzhou, Guangdong, China) using Lipofectamine LTX and PLUS Reagent (Invitrogen Life Technologies, Carlsbad, CA, USA) in GM. After transfection for 24 h, the cells were transferred to DM for continuous culturing.

### 2.7. Plasmid Extraction Was Identified

PMD-19T-miR-423-5p recombinant plasmid (Dalian TaKaRa Biological Engineering Co., Ltd.) and pLV plasmid were double-digested by QuickCut^TM^ Nhe I and QuickCut^TM^ BamHI. The product was purified after electrophoretic detection. For details, see the manual of the Biospin adhesive recovery kit (Bruker Biospin, Fremont, CA, USA). After purification, the double enzyme digestion product was connected with T4 ligase in a water bath at 16 °C overnight; the recombinant plasmid obtained after the connection was transformed and identified and named pLV-miR-423-5p. The C2C12 cells were transfected according to the Lipofectamine LTX and PLUS Reagent instructions, the cells were photographed and collected 48 h later, and the expression of miR-423-5p was detected by qRT-PCR.

### 2.8. Construction of Luciferase Reporter Plasmid

A pair of primers (3′-*SRF*-F and 3′-*SRF*-R) were used to clone the *SRF* 3′ UTR (containing an miR-423-5p binding site) using PCR (Table 1). The PCR products were digested with XhoI and NotI and then cloned into the psi-CHECK^TM^-2 Vector (Promega, Madison, WA, USA). In addition, the recombinant plasmid was named *SRF*-WT. The *SRF*-MUT (without an miR-423-5p binding site) was constructed by mutating the seed region of the predicted miR-423-5p sites by using gene splicing by overlap extension PCR. All constructs underwent verification through sequencing.

### 2.9. Dual Luciferase Reporter Assay

For the luciferase reporter assay, PK15 was cultured in GM. The 50 nM miR-423-5p mimic/miNC were co-transfected with 0.5 μg *SRF*-WT/*SRF*-MUT using Lipofectamine LTX and PLUS Reagent (Invitrogen) in 24-well plates. For each transfection, three separate wells were utilized. After transfection for 48 h, all cells were harvested, and the normalized firefly luciferase activities (ratio of firefly to Renilla luciferase activities) were measured with a Dual-Glo^®^ Luciferase Assay System (Promega).

### 2.10. Real-Time PCR

Total RNA from C2C12 cells was isolated using Trizol reagent (Invitrogen). Following reverse transcription, qRT-PCR was conducted using a 7500 Real-Time PCR System (Applied Biosystems, Carlsbad, CA, USA) and employing a Roche FastStart Universal SYBR Green Master Mix (Roche Molecular Systems; primer sequences are detailed in Table 1). Additionally, β-actin served as the internal control, and differential expression analysis was performed using the relative quantitative ΔCt method.

### 2.11. Enzyme-Linked Immunosorbent Assay (ELISA)

Proteins from transfected cells were isolated and then analyzed by a double-antibody sandwich ELISA kit. The kit included a micro-ELISA strip-plate that had been pre-coated with antibodies specific to SRF. To initiate the assay, standards and samples were pipetted into the designated wells, where they bound to the pre-coated antibodies. After the addition of a horseradish peroxidase (HRP)-conjugated secondary antibody specific for SRF, the strip-plate was incubated, allowing for the formation of an antibody–antigen–antibody ‘sandwich’. Following incubation, any unbound substances were removed during a washing step. Subsequent to the washing, the TMB substrate solution was added to each well, leading to a color change to blue in the presence of the enzyme–substrate reaction. This reaction was specific to the wells that contained the SRF–HRP complex. The addition of a stop solution changed the color from blue to yellow, which was then quantitatively measured by assessing the optical density at 450 nm using a spectrophotometer. The intensity of the color was directly proportional to the amount of SRF present in the samples. By comparing the optical density of the samples to a standard curve, the concentration of SRF in the samples was determined.

### 2.12. Statistical Analysis

All results were analyzed with the SPSS 17.0 software and presented as means ± standard error. The difference between groups was analyzed with Student’s *t*-test. A *p* value below 0.05 was considered to indicate statistical significance, whereas a *p* value below 0.01 denoted high statistical significance.

## 3. Results

### 3.1. Spatiotemporal Expression and Correlation Analysis of miR-423-5p and SRF

**Figure 1:**
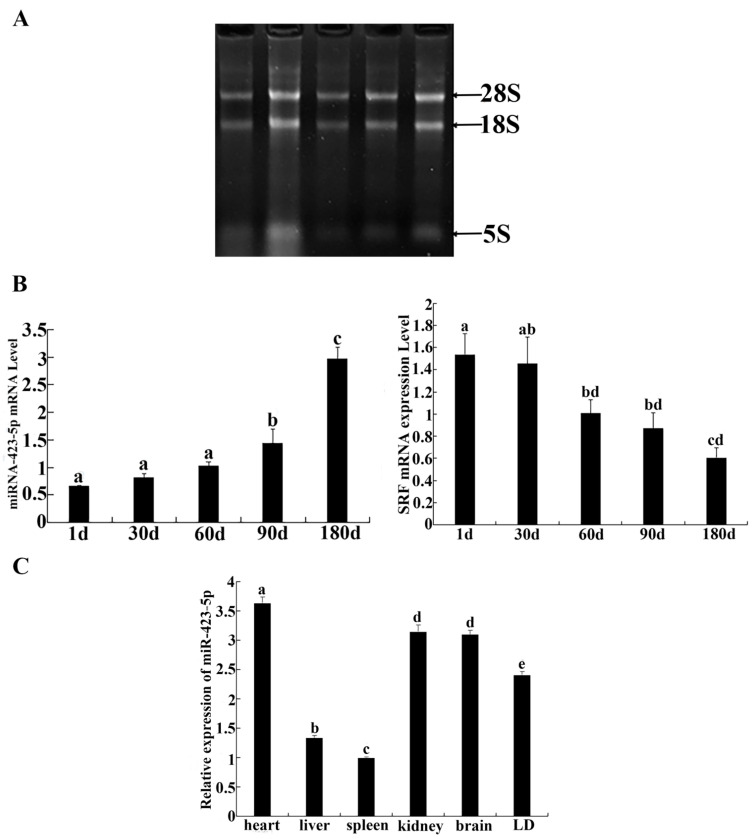
Spatial distribution of miR-423-5p and the serum response factor (SRF): (**A**) Total RNA from skeletal muscle; (**B**) Expression of miRNA-423-5p and SRF in longissimus muscle of *Guangxi Bama miniature pig* during various development periods; (**C**) Expression of miRNA-423-5p in different tissues of *Guangxi Bama miniature pig*. Note: different letters assigned to the values indicate statistically significant differences between the groups (*p* < 0.05). If graphics or values share the same letter, it suggests there was no significant difference between them (*p* > 0.05).

The spatial distribution of ssc-miR-423-5p and *SRF* is shown in Figure 1. The total RNA bands extracted from the skeletal muscle of *Guangxi Bama miniature pig* tissues were complete and clear and could be used in downstream experiments (Figure 1A). The expression profiles of miRNA-423-5p and *SRF* in the longissimus dorsi muscle at 0, 1, 2, 3, and 6 months of age showed that the expression level of miRNA-423-5p increased with the increase in age. No significant difference in the expression of miRNA-423-5p was detected at 1, 30, and 60 days (*p* > 0.05). However, the expression levels at 90 and 180 days were found to be significantly increased compared to those at earlier time points (*p* < 0.05). The expression level of *SRF* in the longissimus dorsi muscle of *Guangxi Bama miniature pig* tissues showed a decreasing trend as age increased (Figure 1B). Next, the SPSS 17.0 software was used to analyze the correlation between *SRF* and miR-423-5p expression. The results showed that the correlation coefficient between *SRF* and miR-423-5p was −0.871. Although *SRF* and Mir-423-5P were negatively correlated, it was not significant (*p* > 0.05). The expression levels of miR-423-5p mRNA differed significantly across the various tissues. The highest expression was observed in the heart, followed by high levels in the kidney, brain, and longissimus dorsi muscle. Conversely, lower levels were detected in the liver and spleen. This information highlights the tissue-specific expression pattern of miR-423-5p (Figure 1C).

### 3.2. Target Prediction of miR-423-5p and KEGG Pathway Analysis

**Figure 2:**
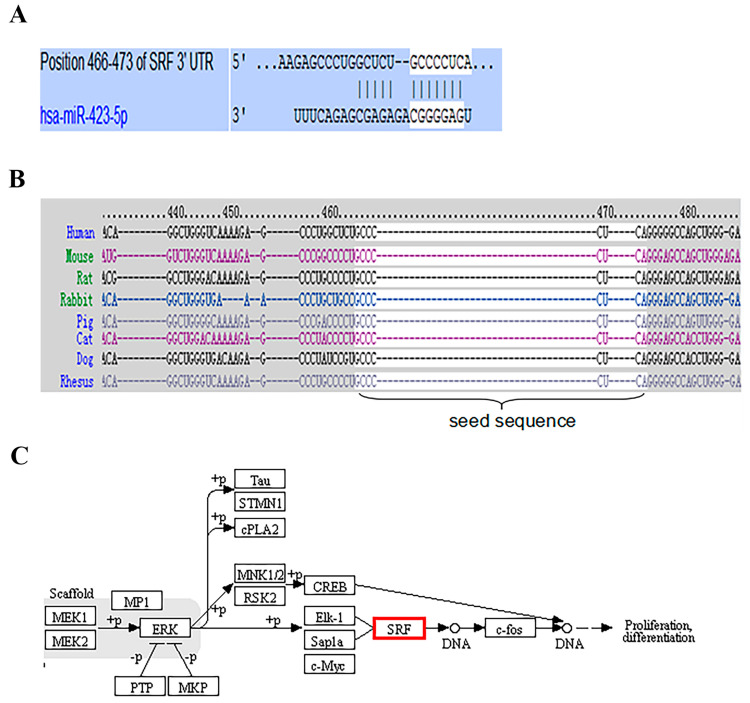
The *SRF* involved in myogenesis and its 3′ UTR had conserved miR-423-5p target sites: (**A**) *SRF* was predicted as a target of miR-423-5p using TargetScan; (**B**) The complementary site for *SRF* and miR-423-5p showed conservation across species; (**C**) Signaling pathways involved in proliferation differentiation of *SRF.*

Using the TargetScan target detection software, *SRF* was identified as a potential miR-423-5p target in humans. A sequence of 8 bp target sites for miR-423-5p (Figure 2A) was detected. The target site between *SRF* and miR-423-5p is highly conserved among mammals (Figure 2B). In addition, the KEGG pathway results showed that *SRF* is involved in myogenesis (Figure 2C).

### 3.3. Construction and Identification of the miR-423-5p Lentivirus Expression Vector

**Figure 3:**
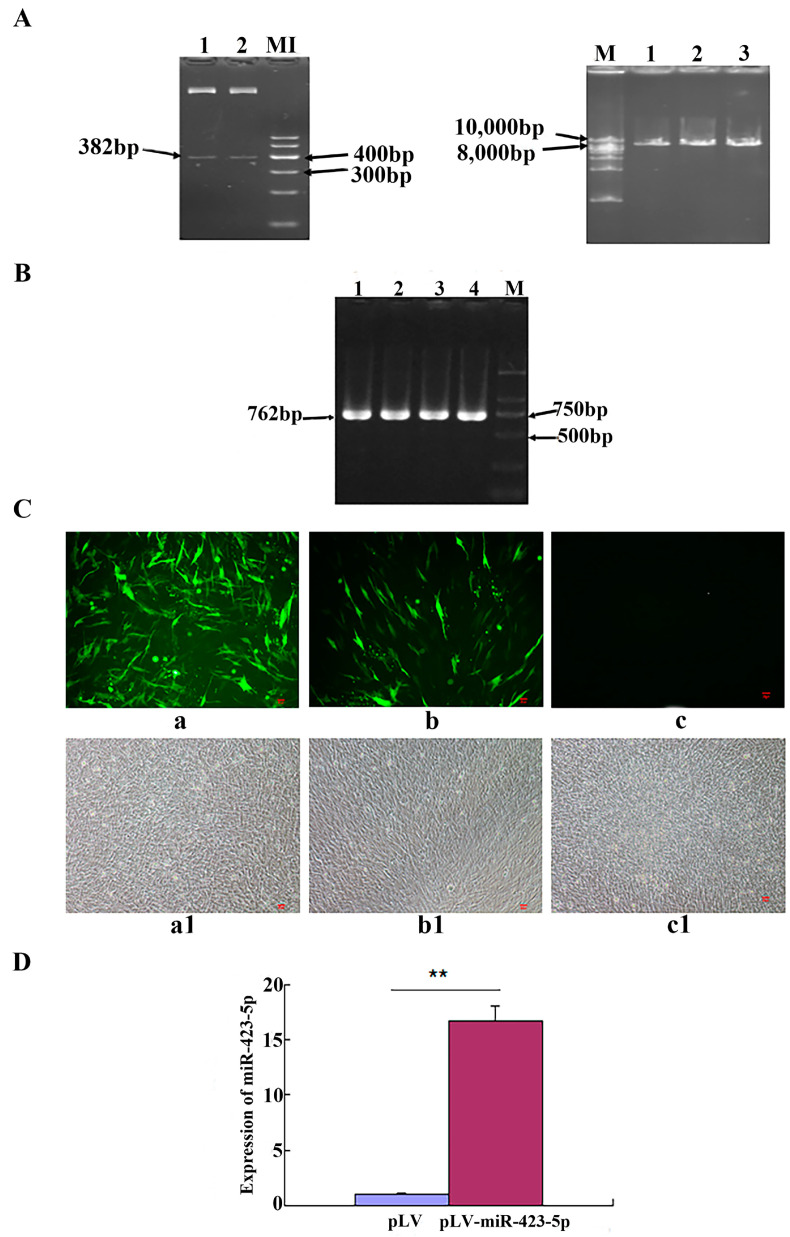
Construction and identification of miR-423-5p lentivirus expression vector: (**A**) Double enzyme digestion products of PMD-19T-miR-423-5p and PLV. Lane 1–2: enzyme digestion products of PMD19-T-miR-423-5p and PLV M1: marker 1. M: 1kb DNA ladder. (**B**) PCR products of pLV-miR-423-5p. Lane 1–4: PCR products of pLV-miR-423-5p M: D2000. (**C**) The functional identification of pLV-miR-423-5p in C2C12 cells. Note: a, a1: pLV-miR-423-5p; b, b1: pLV; c, c1: negative control; a–c: observed by a fluorescence microscope; a1–c1: observed by an optical microscope. (**D**) Expression detection of miR-423-5P in C2C12 cells. Note: ** significant differences (*p* < 0.01).

The PCR results of the product after the double digestion of the PMD-19T-miR-423-5p recombinant plasmid and pLV plasmid showed that the enzyme digestion results of the PMD-19T-miR-423-5p recombinant plasmid had two clear bands, which were consistent with expectations. The results of the enzyme digestion of the pLV plasmid showed a single strip, with a fragment size around 8000 bp, as expected (Figure 3A). The PCR results of the pLV-miR-423-5p recombinant plasmid liquid showed that the strip was single and clear near 750 bp, which was also consistent with the expected result of 762 bp (Figure 3B). Some C2C12 cells transfected with pLV-miR-423-5 plasmid for 48 h were photographed; the results showed that both the experimental (transfected with pLV-miR-423-5p plasmid) and the control (group transfected with pLV) groups showed fluorescence. The results from the experiment demonstrated a successful overexpression of miR-423-5p. The increased fluorescence intensity in the experimental group compared to the absence of fluorescence in the control group (as shown in Figure 3C) indicates effective transfection and expression. Furthermore, the qRT-PCR results obtained 48 h later confirmed this, showing a significantly higher expression of miR-423-5p in the pLV-miR-423-5p-transfected group compared to the pLV control group (Figure 3D). In summary, miR-423-5p was successfully overexpressed.

### 3.4. Identification of the Target Relationship between miR-423-5p and the SRF Gene

**Figure 4:**
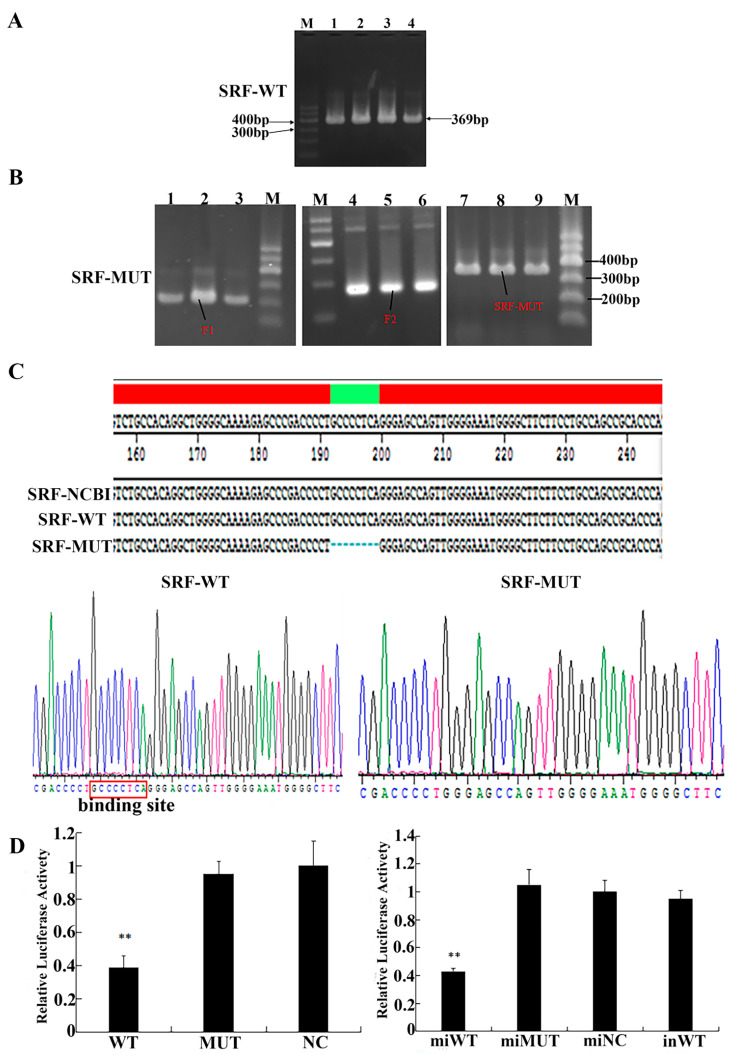
Identification of the target relationship between miR-423-5p and the *SRF* gene. (**A**) PCR products amplified from *SRF*-WT. Lane 1–4: PCR products of *SRF*-WT. M: marker 1. (**B**) PCR products. Lane 1–3: PCR products of F1, Lane 4–6: PCR products of F2, Lane 7–9: PCR products of *SRF*-MUT. M: marker 1. (**C**) Lasergene report of sequencing result. (**D**) Dual luciferase analysis of the interaction between miR-423-5p and target gene *SRF.* Note: ** significant differences (*p* < 0.01).

A bacterial solution was used as the template for PCR verification, and the product was detected by 1% agarose gel electrophoresis. The identification results of the *SRF*-WT recombinant plasmid showed that the band was single and clear, ranging from 300 to 400 bp, which was consistent with the expected result of 369 bp (Figure 4A). The *SRF*-WT recombinant plasmid was used as the template for PCR amplification; the resulting bands were clear, and the fragment size was consistent with the expected results (Figure 4B). The sequencing results showed that the synthesized genes were identical to the sequence recently reported on NCBI. We concluded that the recombinant plasmid vectors *SRF*-WT and *SRF*-MUT were constructed successfully (Figure 4C). To confirm the relationship between *SRF* and miR-423-5p, miR-423-5p mimic/NC and *SRF*-WT/*SRF*-MUT were transfected into PK15 cells. As shown in Figure 4D, the experiment utilizing a luciferase activity assay clearly demonstrated the impact of miR-423-5p on the 3′ UTR of *SRF*. The significant reduction in luciferase activity in the *SRF*-WT-plasmid-transfected PK15 cells co-transfected with the miR-423-5p mimic (miWT group) suggests that miR-423-5p can directly bind to and inhibit the expression of *SRF* via its 3′ UTR. The lack of any effect in the mutant-plasmid-transfected PK15 cells (miMUT) and the reversal of the effect in the *SRF*-WT plasmids co-transfected with the miR-423-5p inhibitor (inWT) further supports this conclusion, indicating a specific interaction between miR-423-5p and the wild-type 3′ UTR of *SRF*.

### 3.5. Functional Validation of miR-423-5p in C2C12 Cells

**Figure 5:**
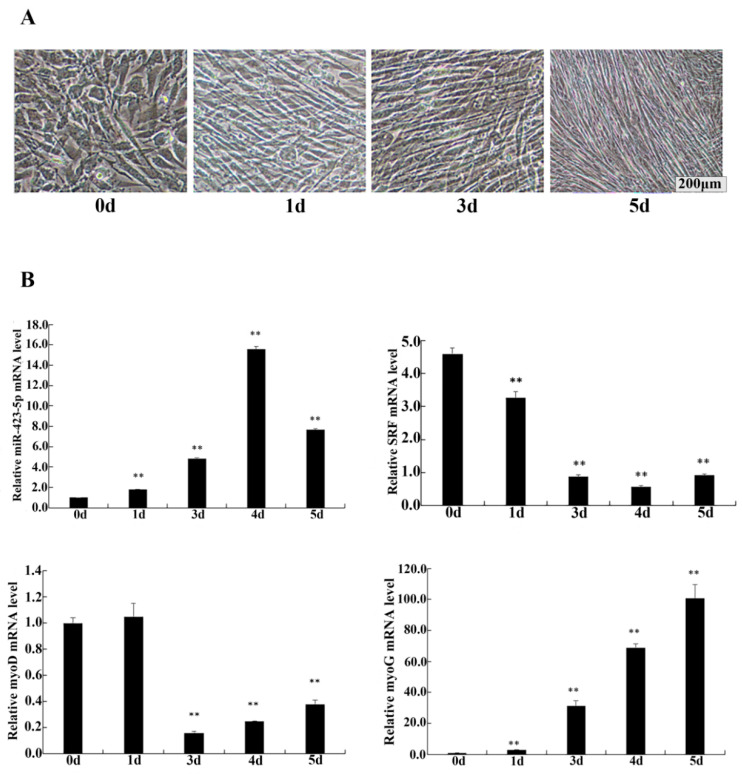
Changes in cell phenotype and expression of related genes during C2C12 cell differentiation were detected. (**A**) Induced differentiation of C2C12 myoblasts. (**B**) Expression of miR-423-5p, serum response factor (SRF), MyoD, and MyoG during C2C12 cell differentiation. Note: ** significant differences (*p* < 0.01).

**Figure 6:**
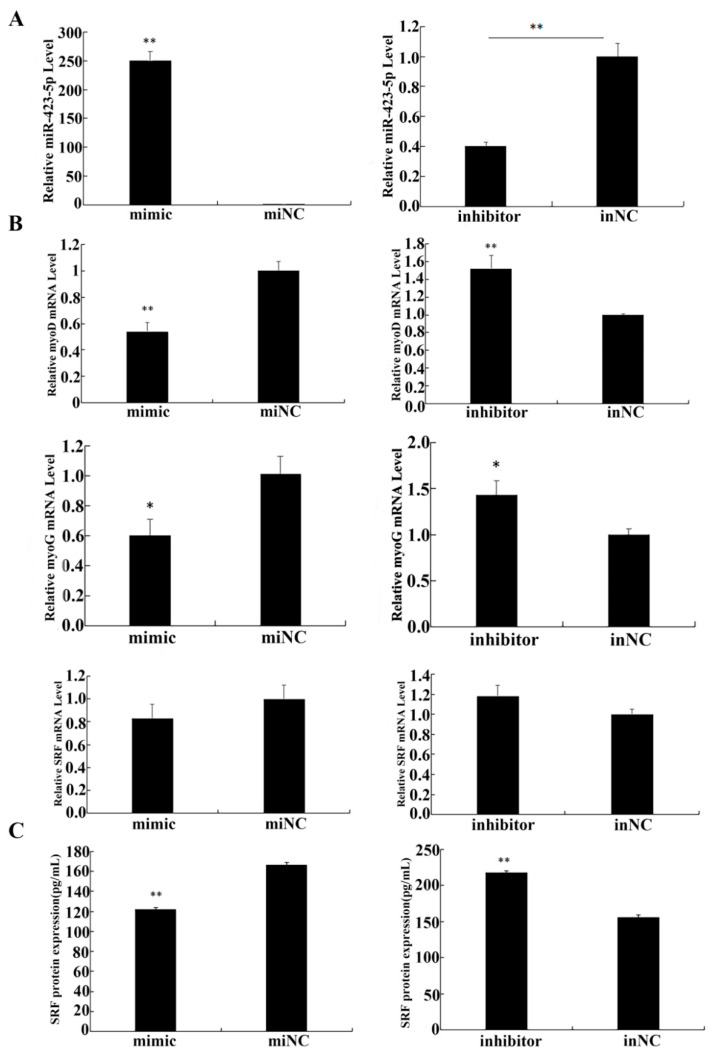
Expression analysis of related genes and proteins after overexpression or inhibition in C2C12 cells. (**A**,**B**) The regulatory role of miR-423-5p, SRF, MyoD, and MyoG in the differentiation of C2C12 cells. (**C**) Serum response factor content determined by ELISA in C2C12 cells treated with miR-423-5p mimic or inhibitor. Note: ** significant differences (*p* < 0.01); * significant differences (0.01 < *p* < 0.05).

To investigate the role of miR-423-5p in myogenesis, C2C12 cells were cultured in differentiation medium (DM) and assessed for the relative expression levels of miR-423-5p, *SRF*, *MyoD*, and *MyoG* using RT-qPCR. The morphological change during C2C12 differentiation was observed by an optical microscope (Figure 5A). The differentiation model was established successfully. The results of the RT-qPCR analysis showed that the endogenous expression of miR-423-5p and MyoG were gradually up-regulated, while the expression of MyoD was constantly up-regulated and then down-regulated, and *SRF* was constantly down-regulated (Figure 5B). After transfection and the induction of differentiation in DM for 2d, in the cells transfected with the miR-423-5p mimic, the expression of miR-423-5p was significantly up-regulated compared to the mock-infected control (miNC) cells (Figure 6A). These changes were aligned with the myogenic markers, MyoD, and MyoG gene mRNA expression, which significantly decreased (*p* < 0.01), as did the *SRF* protein expression. In contrast, the inhibitor group showed a significant increase in the mRNA expression levels of MyoD and *MyoG*, along with an increase in *SRF* protein expression (Figure 6B,C). These results suggested that miR-423-5p plays a negative function in myogenic differentiation by inhibiting the expression of *SRF* protein.

## 4. Discussion

Skeletal muscle is considered an important reference factor for assessing the quality of meat in individual pigs. Its growth and development are closely related to the meat yield of an individual, and the difference in its physiological and biochemical characteristics will directly affect the quality of meat from animals [25]. Skeletal muscle growth and development is a complex process influenced by a variety of genetic and environmental factors. Using the Bama miniature pig strain of individual pigs of different ages and genders, combining molecular and genetic methods can provide valuable insights into the genetic basis of skeletal muscle growth and development. MicroRNA plays a fundamental role in the regulation of myogenesis by regulating a variety of transcription factors and signaling molecules. MicroRNA is not only highly expressed in skeletal muscle but also expressed much more strongly in heart muscle than in the muscles of other organs, which together participate in the regulation of muscle genesis [26]. In the current study, the results of RT-qPCR were similar to previous reports, in that miR-423-5p showed a high level of expression in pig skeletal muscle [27]; the expression level showed a gradual increase with the increase in age [28]. Additionally, we observed that endogenous miR-423-5p was increasingly up-regulated throughout the differentiation of C2C12 cells. Taken together, these findings indicate that miR-423-5p could play a crucial role in the development of skeletal muscle.

MicroRNAs are highly conserved in mammals, and they can negatively regulate gene expression and induce muscle degradation at the post-transcriptional level through the principle of pairing with a complementary base of the 3′ non-coding region (UTR) of target mRNA [29]. Previous studies have reported that miR-208b can mediate skeletal muscle development and homeostasis by specifically targeting TCF12 and FNIP1 [30]. Secondly, miR-155-5p, miR-26a, miR-378, and others can all participate in the proliferation and differentiation of muscle cells by regulating target genes [31,32]. *SRF* plays an active role during cell differentiation, being crucial for both myoblast proliferation and differentiation [33]. Research by Chen indicates that miR-133 promotes myoblast proliferation by targeting and inhibiting *SRF* [34]. In the present study, the dual luciferase reporter assay results indicated that miR-423-5p directly targeted the *SRF* 3′ UTR in swine. To our knowledge, this study is the inaugural report identifying *SRF* as a target gene of miR-423-5p. The sequences of the 3′ UTR of *SRF* and the seed sequence of mature miR-423-5p are well conserved in mammals, implying that the target sites may be important in *SRF* regulation. Moreover, our KEGG analysis results were identical with previous reports about *SRF*, which showed that it is involved in muscle proliferation and differentiation [35,36]. Additionally, our findings indicate that during the differentiation of C2C12 cells, the endogenous expression of *SRF* decreased, while that of miR-423-5p increased. Hence, we speculated that miR-423-5p is involved in skeletal muscle development by directly targeting SRF 3′ UTR.

The myogenic regulatory factors (*MRFs*) *Mrf4*, *MyoD*, *Myf5*, and *MyoG* determine skeletal muscle cell identity. They exhibit a similar regulatory mechanism in muscle-specific expression, yet they function in distinct aspects of myogenesis [37]. Specifically, *Myf5* and *MyoD* specify myoblasts for terminal differentiation in a redundant fashion, whereas *Mrf4* and *MyoG* not only play a direct role in the differentiation process but also induce the expression of myotube-specific genes [38]. In this study, we found that the later expression of *MyoGs* were higher than during the early days during C2C12 cell differentiation. On the contrary, *MyoD* initially increased and then declined. The results were consistent with previous reports showing that *MyoD* has been identified during the early stage of muscle development and that *MyoG* has been proposed to be active in late MRFs [39,40]. In addition, we discovered that *MyoD* mRNA, *MyoG* mRNA, and *SRF* protein level were significantly down-regulated when miR-423-5p was overexpressed. Collectively, these findings indicate that miR-423-5p negatively regulates cell differentiation. Although our study provides important insights for understanding the role of miR-423-5p in muscle cell differentiation, a comprehensive investigation into the regulatory mechanisms of miRNA and *SRF* in porcine skeletal muscle development is still required.

In conclusion, our findings demonstrate that miR-423-5p is abundant in swine skeletal muscles and increases in expression during C2C12 cell differentiation. In addition, *MyoD*, *MyoG*, and *SRF* were significantly down-regulated when miR-423-5p was overexpressed. Moreover, we investigated the nature of the relationship that exists between miR-423-5p and *SRF*; miR-423-5p regulated *SRF* by directly targeting *SRF* 3′ UTR. Collectively, this information indicates that miR-423-5p regulates skeletal muscle differentiation, potentially through targeting *SRF*.

## Figures and Tables

**Table 1:** Primer sequences in this study.

Primers	Primer Sequences (5′→3′)	Application
3′-*SRF*-F	CTCGAGCTCCGTGTTTGCCATGAGTA	3′ UTR amplification
3′-*SRF*-R	GCGGCCGCTTCCCTCCAACCCAGCAG	3′ UTR amplification
3′-*SRF*-DF	GGCAAAAGAGCCCGACCCCTGGGAGCCAGTTGGGGAAATG	3′ UTR amplification
3′-*SRF*-UR	CATTTCCCCAACTGGCTCCCAGGGGTCGGGCTCTTTTGCC	3′ UTR amplification
Q-miR-423-5p-F	AGGGGCAGAGAGCGAGACTTT	RT-qPCR
*MyoG*-F	ACTCCCTTACGTCCATCGTG	
*MyoG*-R	CCAGGGTCTTCTTCATCCGTTC	
*MyoD*-F	GCCTTCTACGCACCTGGAC	
*MyoD*-R	ACTCTTCCCTGGCCTGGACT	
SnU6-F	CGCTTCGGCAGCACATATAC	
SnU6-R	TTCACGAATTTGCGTGTCAT	
Q-*SRF*-F	CAAGATGGAGTTCATCGACAACAAG	
Q-*SRF*-R	CAGTTTGCGGGTGGCAAAG	
β-actin-F	TCCCTGGAGAAGAGCTACGAG	
β-actin-R	GCCGTGATCTCCTTCTGCA	

## Data Availability

All data included in this study are available upon request by contact with the author.

## Abbreviations

miRNAs, microRNAs; miR-423-5p, microRNA-423-5p; SRF, serum response factor; 3′ UTR, 3′-untranslated region; MRFs, myogenic regulatory factors; MyoG, myogenin; RT-qPCR, real-time quantitative polymerase chain reaction; SOE PCR, gene splicing by overlap extension PCR; GM, growth medium; DMEM, Dulbecco’s modified eagle’s medium; DM, differentiation medium; NC, negative control; SEM, standard error; LD, longissimus dorsi.
